# Detainees, staff, and health care services in immigration detention centres: a descriptive comparison of detention systems in Sweden and in the Benelux countries

**DOI:** 10.3402/gha.v9.30358

**Published:** 2016-03-04

**Authors:** Soorej J. Puthoopparambil, Magdalena Bjerneld

**Affiliations:** Department of Women's and Children's Health, International Maternal and Child Health (IMCH), Uppsala University, Uppsala, Sweden

**Keywords:** deportation, health care, detention staff, immigrant detainee, Common European Asylum System, European Union

## Abstract

**Background:**

Immigration detention has been shown to negatively affect the health and well-being of detainees. The aim of the study was to describe and compare policies and practices that could affect the health and well-being of immigrant detainees in the Benelux countries (Belgium, the Netherlands, and Luxembourg) to those in Sweden.

**Design:**

This was a case study. Data were collected in two phases using a questionnaire developed particularly for this study. In the first phase, authorities in the Benelux countries responded to the questionnaire via email. During the second phase, a research team visited detention centres in the Benelux countries to observe and further explore, strengthening findings through triangulation. Data on Swedish detention centres were collected in previous studies.

**Results:**

Compared to the Benelux countries, Sweden has limited health care provision available in the detention centres. Swedish detention centres did not have mental health care professionals working at the centres and had fewer restrictions within the centres with regard to access to mobile phone, internet, and various recreational activities. Compared to Sweden, the detention centres in the Benelux countries have more staff categories providing services to the detainees that are provided with relevant and timely on-the-job training. All the countries, except Belgium, provide subsistence allowances to detainees.

**Conclusion:**

Despite the Common European Asylum System framework, differences exist among the four European Union member states in providing services to immigrant detainees. This study highlights these differences, thereby providing a window on how these diverse approaches may serve as a learning tool for improving services offered to immigrant detainees. In Sweden, the health care available to detainees and training and recruitment of staff should be improved, while the Benelux countries should strive to reduce restrictions within detention centres.

## Introduction

Immigration detention, mainly aimed to facilitate deportation, is widely practised across the European Union (EU) (1). Immigration detention is defined as *the deprivation of an individual's liberty, usually of administrative character, for an alleged breach of the conditions of entry, stay or residence in the receiving country* (2). According to the international and EU guidelines, immigration detention should be avoided to the largest possible extent (3, 4). However, detention persists, and in some countries, such as Sweden, the total number of detainees has increased during the past 5 years (1). The new European Agenda on Migration presented by the European Commission (EC) (5) and the statements issued by the Council of Europe (CoE) (6, 7) emphasise the need for EU member states to reinforce their efforts, including the use of detention, to return migrants who are irregularly (illegally) staying within their territories.

Detaining authorities have the responsibility to safeguard the health and well-being of detainees (3). Yet, studies from around the world have shown the negative impact of detention on the health and well-being of immigrant detainees (8–11). Vulnerable immigrants, such as victims of torture, might be detained (10). Immigration detention creates and exacerbates illness (8, 11) and those effects may last even after immigrants are released from detention (9). However, as long as immigration detention continues to exist, it is important to identify and follow practices and policies that could create a supportive environment to minimise the negative impact of detention on the health of detainees. Supportive environment is of utmost importance for health and such an environment encompasses physical, social spiritual, economic, and political dimensions (12). As stated by the World Health Organization (WHO), *health is created and lived by people within the settings of their everyday life* (13). Several factors such as personal, organisational, and environmental factors within a given setting interact to create a healthy or unhealthy setting (14). The everyday life setting for immigrant detainees is the detention centres; detainees mainly interact with fellow detainees and detention staff. This setting needs to be explored to identify practices that could mitigate negative effects on health. The authors have conducted such explorative studies in the Swedish detention centres (15–17). These studies identified issues, such as language barriers, restrictions within detention centres, limited access to health care, unfavourable staff behaviour, and lack of adequate training and support for detention staff, negatively influencing the health and well-being of detainees.

The present study explored how the aforementioned issues are addressed in selected EU member states. EU member states have similar detention systems as part of the Common European Asylum System (CEAS) which makes them comparable (18). The main policy documents governing conditions of immigration detention in all EU member states are the *Reception conditions directive* (19) and the *Return directive* (4). Articles 10 and 11 of the *Receptions conditions directive* and articles 16 and 17 of the *Return directive* lay down the minimum standards that need to be ensured while detaining immigrants. The CoE (20), the European Committee for the Prevention of Torture and Inhuman or Degrading Treatment or Punishment (CPT) (21), as well as other international organisations have also issued guidelines to ensure the humane treatment of immigrant detainees (3, 22). The directives and guidelines state requirements that include keeping immigrant detainees separate from ordinary prisoners; giving detainees access to open air spaces; providing information to detainees; explaining the rights and rules to be followed, in a language detainees understand or are reasonably expected to understand; providing access to appropriate medical care, and screening for physical and mental health on arrival; and providing access to recreational and educational activities. The three EU member states chosen to be compared with Sweden were Belgium, the Netherlands, and Luxembourg (together known as the Benelux countries). Practicalities such as getting access to the detention centres and limited resources restricted the comparison to three countries. Detention centres in the Benelux countries and Sweden were found to be more comparable, when compared to other EU member states. For example, in the United Kingdom, there is no maximum time limit for detention (23), and in Germany, detention practices vary among the federal states (1).

## Aim

The aim of the study was to explore and describe policies and practices that could affect the health of immigrant detainees in the Benelux countries compared with those in Sweden. Practices and policies that could affect the health of detainees were identified based on the results from the studies conducted in Swedish detention centres (15–17) and the international guidelines (3, 20, 21). The comparison focused on the living conditions of detainees, their access to health care in detention centres, the categories and training of staff working at the detention centres. Other influences, such as legal and political conditions, affect the health and well-being of detainees, but we restricted our comparison to the aforementioned attributes. The comparison did not focus on any health outcome indicators across the countries.

This study is part of a larger project aimed at identifying factors that could mitigate the negative effects of immigration detention on the health and well-being of detainees.

## Methodology

This was a descriptive case study comparing current immigration detention policies and practices in the four countries (24). Case studies are helpful when an extensive description of a phenomenon is required (25). Yin suggests structured interviews (‘in lines of a formal survey’) and observations as suitable data collection methods for case studies (25). Although we did not conduct interviews in the traditional sense, that is to say, individual in-depth or semi-structured interviews, we collected data using a structured questionnaire sent via email to representatives of the governmental agencies responsible for running immigration detention centres in the Benelux countries. The data obtained were further corroborated through observations and discussions during visits conducted to detention centres in these countries.

To facilitate triangulation, data were collected in two stages using multiple investigators and data sources (25, 26). Data were collected using a questionnaire developed based on our previous research (15–17) and other international detention monitoring tools (1, 2, 27). The questionnaire included questions concerning staff training arrangements, the categories of staff working with detainees, daily subsistence allowances for detainees, food serving schedules, medical services, interpretation services, and access to recreational and educational activities at the detention centres. Face validity (26) of the questionnaire was established through discussions with relevant experts working with immigration detention, in Sweden and other EU member states. The expert group consisted of members from non-governmental organisations (NGOs), the Swedish border police, and researchers.

During the first stage of data collection, the questionnaire was emailed to representatives, one representative from each country, of the governmental agencies responsible for immigration detention in the Benelux countries. These representatives hold senior level positions at the agencies. Completed questionnaires from all the countries were obtained prior to the visits. During the second stage, a research team consisting of the researchers (the two authors), a representative from the International Organization for Migration (IOM), and a representative from the Swedish Migration Agency (SMA) visited detention centres in the Benelux countries. The representatives did not participate in the questionnaire development, the selection of the countries or detention centres to be visited, or the data analysis and drafting of this article. However, their participation in the visits and data collection (through observation) contributed to triangulation through the use of multiple investigators who have several years of experience working with various aspects of immigration detention in the EU. The aim of the visits was to further explore and observe, and thus to triangulate the information provided by the authorities.

The team visited all detention centres in the Netherlands (three) and Luxembourg (one), and two out of five centres in Belgium. During each visit, the detention centre's management team briefed the research team on the centre's rules and practices. The research team discussed and clarified any issues related to the answers provided in the questionnaire. After the briefing, the research team was given a guided tour of the centre and its facilities, which enabled the team members to observe the living conditions of detainees and other aspects mentioned in the questionnaire. Every day, after the visits, the research team met and discussed their observations. The first author wrote field notes during these meetings and the visits, which complemented and corroborated (26) the data obtained from the questionnaire. After each country visit, all members in the research team answered the same questionnaire, separately, based on their observations and information obtained during the briefings. This organised the research team's observations in the same format as that of the questionnaire, facilitating triangulation and easier comparison. Triangulating the information obtained from the authorities via email with observations by the research team members increased the rigour of the collected data and the research process.

On the Swedish side, the representative from the SMA answered the questionnaire via email. The answers provided by the representative confirmed the information available to the authors from the data collected for the previous studies (15–17). Separate visits to the Swedish detention centres were deemed unnecessary at this time since the researchers had visited all the Swedish detention centres multiple times for data collection for their previous studies.

In order to validate the results (24, 25), the study results were both presented at a follow-up meeting and sent to the representatives from the four countries and were confirmed to be accurate depictions.

### Ethical considerations

This study deals with the policies and practices followed in immigration detention centres in four countries and does not report any research or experimentation with human subjects. The data collected in the study were on existing public policies governing immigration detention. No personal information, opinions, experiences, or any other information that could be used to identify any individual were collected. No personal information about the individuals who cooperated with the study will be revealed at any point. Prior to being invited to participate in the study, the representatives of the public agencies in the Benelux countries were informed about the aim of the study, the potential use of the results, and the authors’ intention to visit the centres. Once they agreed, the questionnaire was sent to them. The studies from which the data on the Swedish system were obtained (15–17) have ethical approval from the regional ethical review board in Uppsala (Dnr 2011/463), Sweden. These studies required ethical approval as data concerning personal experiences were collected through in-depth interviews with Swedish detention staff and detainees.

## Results

The results are divided into four sections describing and comparing policies and practices followed in the immigration detention centres in the four countries. The first section gives an organisational overview of the detention system followed by sections describing detainees’ living conditions, staff working with detainees, and detainees’ access to health care.

### General characteristics

Table 1 provides details on the general characteristics of detention systems in the four countries. Government agencies manage and run detention centres in all the countries. However, in Luxembourg, a private company is entrusted with the external security of the centre. They also accompany detainees, along with detention staff, during hospital visits as well as when detainees are taken to activity rooms outside their living areas. Belgium was the only country where detainees did not receive any financial subsistence allowances, but if they helped with housekeeping tasks, such as cleaning and laundry, they could receive vouchers worth one to five Euros, which could be used at shops at the centres. However, there were not enough tasks for everyone to benefit from this allowance. None of the countries used prisons for immigration detention, but immigrants who had committed crimes, completed their prison sentences, and were waiting to be deported were sometimes detained in immigration detention centres along with other immigrants. In other instances, they were directly deported from prisons. For example, in Belgium, in 2014, a total of 272 immigrant prisoners, who had finished their prison sentences, were transferred to detention centres to be deported, and 353 immigrant prisoners were deported directly from prisons.

**Table 1:** General information on the detention system in the four countries (as of 2014)

	Belgium	Luxembourg	Sweden	The Netherlands
Are private companies allowed to run detention centres?	No	No	No	No
Number of centres	5	1	5	3
Total capacity	481	88^a^	255	1,522
Maximum allowed duration of detention	18 months	12 months	12 months	18 months
Average duration of detention	34 days	27 days	8^b^ days	72 days
Total number of detainees in 2014	5,601	392	3,201	2,821
Detainee allowance/week	0€	21€	~17€	15€
Average cost per detainee/day	186€	425€	410€	200€
Total cost for detention (in million)	~35.4€^c^	~4.3€	~36.4€	~130€

a Although some of the detention rooms were built to accommodate two adults, the management found it not spacious enough for two adults to be accommodated for longer periods. In practice, the total capacity of the centre is 44, i.e. one detainee per room.

b Annual report SMA, 2015, p. 100 (28).

c In 2013, Data from EMN report, p. 44 (1).

### Living conditions

As depicted in Table 2, all four countries use similar methods to communicate with detainees who do not speak one of the common languages at the centres. All countries, except Sweden, provide detainees with written information (usually in the form of brochures) regarding the rules and regulations to be followed in the centres, in a language assumed to be understood by detainees. Detainees in Swedish detention centres are mainly informed verbally about the rules and regulations. Additionally, short written information on the rules and regulations in two or three languages, such as English, Swedish and Arabic, is displayed on the notice boards at the centres. In Belgium, detainees go through a three-stage intake process on arrival. During the first stage, a social worker explains to detainees their legal cases and the basis for their detention. During the next stage, a staff member belonging to the educator staff category, a category of staff responsible for social and practical aspects of detainees’ lives, informs detainees about various services available and rules to be followed in the centre. Finally, a nurse performs medical screenings. Additionally, detainees are shown a DVD explaining the rules of the centre, the repatriation process, and other relevant information.

**Table 2:** Living conditions in detention centres

	Belgium	Luxembourg	Sweden	The Netherlands
Detainees per room	2–30	1–2	2–5	2
Are detainees locked up in their rooms during night?	No	Yes	No	Yes
Minimum access to courtyard (per day)	2 hours	Anytime during the day, except during lunch	3 hours	1 hour
Unrestricted access to activities at the centres	No	No	Yes	No^a^
Interpretation services	Interpreter/staff/fellow detainees	Interpreter/staff/fellow detainees	Interpreter/staff/fellow detainees	Interpreter/staff/fellow detainees
Access to internet	No^b^	Restricted	Unrestricted	Restricted
Is use of mobile phones allowed?	Yes	No	Yes	No
Staff categories who come in regular contact with detainees	Medical staff Social worker Educator Security Management	Medical staff Social worker Security staff Administrative staff	Supervisor Case officer	Medical staff Supervisor Activity coaches/sports instructors Religious counsellors, social workers

a The centre in Rotterdam offers unrestricted access to activities.

b The centre in Vottem grants internet access (except Skype) to detainees for an hour. Authorities reported that a project aimed at providing internet access at all centres is underway.

Internet and mobile phone use was restricted in all countries except Sweden. In the Netherlands, detainees were only allowed to access websites permitted by the authorities. They did not have access to communication services such as email, Facebook (29), or Skype (30). In Luxembourg, only Skype was unavailable. In Sweden, detainees were provided with a mobile phone without a camera if they did not already have one. No mobile phones were allowed in the Netherlands and Luxembourg. In Belgium, detainees could use their mobile phones if the phones lacked cameras, borrow mobile phones for a short time from the staff, or buy phones from the shop at the centre. All the countries, except Sweden, offered restricted access (1–2 hours per day) to various activities such as access to internet, library, and gym at the detention centres. Detainees are locked in during the night in Luxembourg and the Netherlands.

Belgium and the Netherlands regularly conducted surveys among detainees regarding satisfaction on the services provided at the detention centres.

### Detention staff

The security staff members (also known as supervisors in the Netherlands) in the Benelux countries wear uniforms. No staff members in Sweden wear uniforms. In Belgium and Sweden, repatriation staff working on detainees’ deportation cases were employed at the centres, and they regularly interact with detainees. In Luxembourg and the Netherlands, the repatriation staff is not part of the detention staff.

Training in basic aspects, such as first aid and fire extinguishing techniques, are offered to staff in all the countries. Furthermore, the detention staff in the Benelux countries attend mandatory training on topics such as intercultural communication, nonviolent communication, and self-defence. Similar training is offered to Swedish detention staff, but they are not mandatory and it may take a while (about 1–2 years) before they have the opportunity to attend such trainings. In the Netherlands, the staff members usually attend the training within 6 months of their employment.

### Access to health care in detention centres

As indicated in Table 3, Sweden does not offer any entry or exit medical screening at the centres. All the countries, except Sweden, have mental health care professionals working at the centres. If nurses visiting the Swedish detention centres deem it necessary, detainees can be referred and taken to a mental health professional at the local health centre. In Sweden, county councils in which the centres are located are responsible for providing health care services to detainees. In the Benelux countries, health care providers are employed at the centres or visit the centres regularly, according to agreements with local hospitals. Table 3 shows the limited health care services available in the Swedish detention centres, compared to those in the Benelux countries. For a medical visit outside detention centres, detainees in Sweden have to pay approximately five Euros at the health care centre, even if the visit is a referral from the nurse at the centre. If the detainees report not having money to pay, SMA bears the cost. Detainees in the Benelux countries do not pay for referral health care visits outside the detention centres.

**Table 3:** Health care provisions in detention centres

	Belgium	Luxembourg	Sweden	The Netherlands
Health care access for detainees	As required	As required^a^	Access to care which cannot be deferred^b^	Same as citizens
Daily access (5 days a week) to a nurse	Yes	Yes	No^c^	Yes
Regular access for detainees to doctor at the centres	Yes	Yes	No^d^	Yes
Regular access for detainees to a psychologist at the centres	Yes	Yes	No^e^	Yes
Do health care professionals receive training to work in immigration detention centres?	Yes	No	No	Yes^f^
Entry/Exit medical screening for detainees	Yes/Yes	Yes/No	No/No	Yes/Yes

a Dental care: only emergencies are treated and paid by the centre.

b Detainees also have right to maternal care, care related to abortion, and contraceptive advice.

c Only one centre has a nurse a visiting the centre 5 days a week. The rest of the centres have nurses visiting twice a week.

d Only one centre has a doctor visiting the centre once a week. In the other centres, if found necessary by the nurse, detainees are taken to a health care facility for consultation with a doctor.

e At one of the centres, if found necessary by the nurse, a counsellor can visit the centre for half a day once a week.

f Nurses get training to be a judicial nurse at the training institute run by the Custodial Institutions Agency.

None of the countries, except Belgium, provides any special training to health care professionals at the detention centres. In Belgium, health care staff, such as the nurses employed at the centres, receive the same basic training as other detention staff in aspects such as intercultural communication and dealing with aggression. The Belgian health care staff can request specific trainings, but the authorities reported that such trainings are not easy to find. Detention centres in the Netherlands are run by the Custodial Institutions Agency and nurses in the detention centres receive training to work as judicial nurses, that is, training to work in prisons. Health care professionals in all the four countries are allowed to use interpreters during medical consultations. However, it was reported during the briefings that fellow detainees were occasionally used as interpreters during medical consultations.

## Discussion

The results describe and compare policies and practices in the Benelux countries with those in Sweden, which could affect the health of immigrants in immigration detention centres. This section discusses the results in light of the EU directives (4, 19), international guidelines (20, 22), and studies conducted around the world.

Access to health care at the detention centres varied considerably between Sweden and the Benelux countries. Detainees in Sweden had comparatively less access to health care. Within the EU, Sweden has one of the most restrictive health care provisions for asylum seekers and irregular migrants (31, 32), categories that include the majority of the detainees in Sweden (17). Lack of access to adequate medical care in immigration detention centres has been found to result in increased morbidity and mortality (11, 33–35). Though recommended by international organisations (3, 22), Swedish detention centres lack entry and exit medical screening. All newly arrived asylum seekers in Sweden are offered medical screening. However this is voluntary and, in 2014, only 44% of the newly arrived migrants undertook the screening (36). Detainees in Sweden tend to spend an average of 31 months in Sweden before being detained (17). During this period, before being detained, their health status may worsen as a result of limited access to medical care and social services (31, 32), and suboptimal living conditions due to their legal status (37, 38). This highlights the need to ensure adequate health care for this vulnerable group, at least while they are detained. Medical screening on arrival helps detaining authorities to identify and provide necessary care for detainees who otherwise have limited opportunities to access health care. Medical screening would also protect detention staff and fellow detainees from contracting contagious diseases. The results show that the Benelux countries are taking steps to address the health needs of detainees, whereas Sweden has not yet adequately addressed the issue. Studies show that detainees suffer from mental illness (9, 10) and the need to offer mental health care is therefore evident. This need has been highlighted by detention staff and detainees in Sweden (15, 16). In a recently concluded study assessing the quality of life (QOL) of detainees in the Swedish detention centres, detainees scored the lowest in the psychological domain (scored 41.9/100) (17). In 2009, Sweden was criticised by the European Committee for the Prevention of Torture and Inhuman or Degrading Treatment or Punishment (CPT) for lack of provisions for mental health care and medical screening on arrival at the detention centres (39). The standards recommended by the CPT in relation to immigration detention clearly states the need to *promptly examine all newly arrived detainees by a doctor or by a fully-qualified nurse reporting to a doctor* (p. 71) and *at a minimum, a person with a recognised nursing qualification must be present on a daily basis at all centres for detained irregular migrants* (p. 73) (21). Yet, Swedish detention centres lack regular professional mental health care services and medical screening on arrival.

Another major difference between Sweden and the Benelux countries is the array of staff categories providing services to detainees. Earlier studies have shown the difficulties experienced by staff, such as emotional dilemmas and fear of being assaulted by detainees (16, 40). The Swedish detention staff found it emotionally difficult to integrate the task of facilitating repatriation while providing humane care for detainees (16). The division of roles between different categories of staff, similar to those in the Benelux countries, might help in addressing these challenges (41). The Benelux countries have different, specialised staff categories to address, for example, the security, legal, and social aspects of detention. All categories of personnel work together, but have unique responsibilities, with a clear division of roles and duties. In the Netherlands and Luxembourg, staff working with the legal aspects of repatriation are not part of detention staff and do not work at the centres. Such a system might be worth exploring to address the emotional dilemmas experienced by the Swedish staff. The importance of on-the-job training cannot be underestimated. Compared to the Benelux countries, Swedish staff receive less training. The Swedish system could benefit from the example of the Benelux countries in providing timely and customised trainings such as cross-cultural and nonviolent communication. The EU directives fall short of effectively addressing the training requirements for detention staff working with immigrants who are not convicted of any criminal offenses, yet detained, and who might have been tortured or are sick (4, 19). However, guidelines issued by the CoE (p. 34) and the CPT (p. 66) recognise the need and emphasis on careful recruitment and appropriate training for the staff in detention centres (20, 21). The guidelines recommend that, in addition to the training in interpersonal communication skills and cultural understanding, detention staff should be provided with training to recognise stress reaction symptoms and take appropriate action. According to the results, none of the four countries seems to have such a comprehensive training program for detention staff. Such training programs are needed to safeguard the health of detainees.

Studies conducted among health care staff providing services to immigrants in detention centres (42, 43) and other settings (44, 45) have shown that they face personal and professional challenges. There is a clear lack of customised training for health care providers working with immigrant detainees. Health care providers working in detention also need training to provide culturally competent care for the varied needs of this specific group. The literature clearly shows that health care providers with cultural competence as well as intercultural communications skills are a critical factor in successful and appropriate health care interventions and patient receptivity to care (46–48).

Clear communication is essential for identifying mental health issues since immigrants, depending on their culture, have different ways of expressing their mental health concerns (47). This communication becomes challenging and results in suboptimal care if detainees and health care providers do not speak a common language and do not use professional interpretation services (48, 49). Health care providers at detention centres in all the four countries use professional interpreters. However, the occasional use of fellow detainees as interpreters during medical consultations is a clear breach of confidentiality and may subsequently prevent a sick detainee from disclosing important health-related information (50, 51). The discussion of subjects such as homosexuality, sexual violence, sexually transmitted infections, or communicable diseases can be highly sensitive, particularly in the close quarters of multicultural detention centres.

Detainees’ inability to acquire and understand information related to their situation in detention has been shown to cause uncertainty and negatively affect their health and well-being (15, 17). Language barriers, again, pose challenges to availability of and access to information. In a recent study conducted among immigrant detainees in Sweden, one of the detainees reported that, often, not being able to understand the content of the documents that he was required to sign caused him stress and led to his distrust of authorities (15). Another study conducted among health care professionals working with asylum seekers in Sweden reported that migrants might not come for health screenings because they were unable to read and understand the information about the screenings, which was in Swedish (44). Studies have also highlighted difficulties in accessing professional interpreters when needed, making the information delivery delayed or suboptimal (15, 44, 49, 51). The provision of a booklet containing general information and rules in a language detainees are presumed to understand, as practiced in the Benelux countries, is much needed in Sweden, in addition to spoken information. This recommendation is in line with the provisions in the EU directives and guidelines (4, 19–21), whereby the member states are required to provide information regarding legal decisions and other rules relevant to the detainees’ situation in a language reasonably supposed to be understood by detainees. CPT recommends that detainees should be provided with a document explaining detainees’ rights, simply and clearly, in a language commonly spoken by detainees (21).

Detention is a restriction on individuals’ freedom of movement and liberty. Additional restrictions within detention are not advisable as they negatively affect the health and well-being of detainees by creating a feeling of imprisonment (15). Compared to the Benelux countries, Swedish centres have limited restrictions within the detention centres. Detainees are not locked in at night, they have unrestricted access to various activities and communication channels, which is an important link to their family and friends as well as to other information sources. A less restrictive environment within immigration detention centres and other similar confined facilities have been shown to have positive effect on inmates’ well-being (15, 52).

### Methodological limitations

This study was conducted during early 2015. Any changes implemented in the countries thereafter are not reflected on here. The chances of data collected during the visits being influenced by the presence of representatives from the SMA and IOM are limited since the aim of the visits was to corroborate the initial set of data, which was collected prior to the visits. Moreover, the data were collected on existing policies and not on aspects such as personal experiences, which could be greatly influenced by the interaction between data collectors and informants. No causal inference can be drawn because this was a descriptive case study. The seemingly better policies, such as increased access to health care in detention centres in the Benelux countries, might not result in better health for detainees because policy implementation gaps may exist. Such gaps, if any, were not assessed as part of this study. This study is a descriptive comparison of selected aspects of a complex system involving various stakeholders. We have only managed to obtain the policies and practices described by the authorities. Acquiring responses from detainees, detention staff, and NGOs could, for example, have provided a more detailed view of the situation. Due to practical challenges and limited resources, we restricted our data collection to policies. However, through triangulation (data source and investigator) we aimed to strengthen the validity and reliability of the results (25) and the study design (26). Results from this study, with restricted scope, have limited comparability to the results from the explorative in-depth studies conducted in Sweden (15–17), which prompted this study. For example, even if the detainees in Benelux countries seem to have better access to health care in detention, we cannot conclude that detainees in the Benelux countries have better health than detainees in Sweden since we did not compare health outcomes or personal experiences of detainees across the four countries. The same holds true for other aspects such as staff training. Although the Benelux countries seem to have a more systematic approach in training their staff, the effectiveness of such trainings cannot be assessed by the current study. Irrespective of the methodological limitations, this study provides a unique investigation into the policies and practices in immigration detention centres in the four countries.

## Conclusion

Even within the common framework, the CEAS, the results show the diversity in the provision of services for immigrant detainees. All the four member states in this study can improve their services at the detention centres, thereby potentially contributing to efforts to mitigate the negative impact of detention on the health of immigrant detainees. Detention staff, including health care professionals working in the detention centres, require customised training appropriate to the needs of working with immigrant detainees. The health care, especially mental health care, available in Swedish detention centres must be improved. The training and recruitment of detention staff and information delivery to detainees could be substantially improved. Restrictions within the centres in the Benelux countries, especially access to communication channels, should be avoided as much as possible. By investigating the diversity of approaches, this study provides a way forward for immigration detention centres to explore measures to minimise the negative impact on the health of detainees. The results can also be relevant to other EU member states since the CEAS is applicable to all member states.
